# Anemia and its association with coffee consumption and hookworm infection among pregnant women attending antenatal care at Debre Markos Referral Hospital, Northwest Ethiopia

**DOI:** 10.1371/journal.pone.0206880

**Published:** 2018-11-08

**Authors:** Gemechu Kumera, Kalkidan Haile, Nurilgn Abebe, Tefera Marie, Tewodros Eshete

**Affiliations:** 1 Department of Public Health, College of Medicine and Health Sciences, Debre Markos University, Debre Markos, Ethiopia; 2 Debre Markos Referral Hospital, Debre Markos, Ethiopia; 3 Department of Midwifery, College of Medicine and Health Sciences, Debre Markos University, Debre Markos, Ethiopia; 4 Department of Health Informatics, College of Medicine and Health Sciences, Debre Markos University, Debre Markos, Ethiopia; National Institute of Health, ITALY

## Abstract

**Background:**

Anemia in pregnancy is a major public health concern worldwide, especially in developing countries. Thus, there is a need of having current information and local data on the prevalence of anemia and associated factors during pregnancy to help inform preventive programmes. The aim of this study was to assess the prevalence of anemia and associated factors among pregnant women attending antenatal care at Debre Markos Referral Hospital, Northwest Ethiopia.

**Methods:**

An institution based cross-sectional study was conducted at Debre Markos Referral Hospital in July and August 2016. A total of 234 randomly-selected pregnant women took part in the study. Data on sociodemographic factors, environmental and sanitation factors, reproductive factors, and nutrition related characteristics were collected using a structured questionnaire. Hemoglobin level was determined using hematological analyzer (Cell Dyn 1800) machine. The stool sample was collected to identify intestinal parasitic infections. Statistical analysis was done using logistic regression. The p value of less than 0.05 at 95% confidence interval was considered statistically significant.

**Results:**

The overall prevalence of anemia among pregnant women was 11.5% (95% CI: 8.2%– 14.9%). The result of multivariable analysis revealed that, coffee consumption [AOR = 2.91; 95% CI (1.63, 8.78)], and hookworm infection [AOR = 2.65; 95% CI (1.48, 4.72)] were factors significantly associated with anemia among pregnant women.

**Conclusion:**

Anemia is of public health concern among pregnant women in the study area. All pregnant women coming to antenatal clinics should be screened and treated routinely for intestinal parasitic infection. Pregnant women should limit coffee consumption, and avoid drinking coffee with meals.

## Background

Anemia in pregnancy is a major public health concern worldwide, especially in developing countries[1, 2]. Anemia is characterized by a decline in the concentration of circulating erythrocytes or hemoglobin (Hgb) and impairment in the oxygen transporting capacity[3]. The World Health Organization (WHO) defines anemia in pregnancy as a hemoglobin levels less than 11g/dl[4]. According to WHO, anemia is considered of a public health concern if the prevalence of anemia is 5% or higher[2]. Anemia in pregnancy affects almost half of all pregnant women (41.8%) globally[2, 5]. The WHO estimated that anemia affects about 56% of pregnant women in low and middle income countries, with the highest prevalence in Africa [2, 6–8].

The causes of anemia during pregnancy are multifactorial and include inadequate dietary intake leading to deficiencies in iron, vitamin B12, folate and vitamin A, increased iron requirements due to physiological demands during pregnancy, poor iron bioavailability, intestinal parasitic infections, malaria, hemoglobinopathies, chronic infections like TB and HIV[9–17]. In Sub Saharan Africa inadequate intake of diets rich in iron is shown to be the main cause of anemia among pregnant women[13].

A number of studies suggest possible adverse effects of maternal anemia on pregnancy and child outcomes, including prolonged labor, postpartum hemorrhage, intrauterine growth restriction, intrauterine deaths, preterm delivery, low birth weight, low APGAR score, fetal anemia, increased perinatal mortality, and impaired physical and cognitive development of children[6, 18–23]. Anemia during pregnancy has also been associated with adverse health effects for mother, including fatigue, reduced work productivity, impaired immune function, and increased risk of maternal morbidity and mortality[2, 6, 23, 24]. Some studies have shown that anemia is responsible for about 20% maternal deaths in Africa[25].

Ethiopian Demographic and Health Surveys(EDHS) 2011 reported that 22% of pregnant women were anemic[26]. Other studies conducted in different parts of Ethiopia also reported high prevalence of anemia among pregnant women[27–34]. All the studies consistently indicated the public health significance of maternal anemia in the country. The government has strengthened different interventions to reduce the burden of maternal anemia. However, the outcome of these interventions has not yet knocked mitigating effect on the prevalence of maternal anemia in Ethiopia. In Ethiopia, a number of studies assessed anemia and its determinants, and came up with a significant variation in prevalence of anemia, and divergent and equivocal risk factors. Therefore, there is a need of having current information and local data on the prevalence of anemia and associated factors during pregnancy to help inform preventive programmes. Thus, this study aims to determine the prevalence of anemia and associated factors among pregnant women attending antenatal care at Debre Markos Referral Hospital, Northwest Ethiopia.

## Methods

### Study design and setting

An institution based cross-sectional study was conducted at Debre Markos Referral Hospital in July and August 2016. Debre Markos Referral Hospital is situated at Debre Markos town, East Gojjam administrative zone, 300 km from Addis Ababa, the capital city of Ethiopia. The hospital provides health service to over 3.5 million populations in its catchments, the hospital has 132 beds for inpatients service and the hospital also provides health services for outpatients. Debre Markos town lies on the average at 2, 630 meters above sea level. Over 100,000 populations reside in the town[35, 36]. The study populations were all pregnant women who attend antenatal care (ANC) at Debre Markos Referral Hospital.

### Sample size and sampling technique

Single population proportion formula was used in determining adequate sample-size for estimating the prevalence of anemia. The sample-size of 234 pregnant women was computed with the assumptions of 95% confidence level, 5% margin of error, 16.6% expected prevalence of anemia[34], and 10% non-response rate. Systematic random sampling technique was used to select the study subjects from antenatal clinic during the data collection period. According to the Hospital report, on average, 30–40 pregnant women visit the ANC daily, and 804 pregnant women have been enrolled to ANC at the Hospital. Since the sample size was determined as 234, a sampling interval of 3 was used to select study participants. Of the first three pregnant women, one woman was randomly selected by using lottery method. Eventually, every 2^nd^ pregnant women were selected to participate in the study until the required sample size of 234 pregnant women was obtained.

### Data collection methods

#### Questionnaire

Data on sociodemographic factors, environmental and sanitation factors, reproductive factors, and nutrition related characteristics were collected using a structured and pretested questionnaire through a face to face interview. The section of the questionnaire on dietary diversity was adopted from Food and Nutrition Technical Assistance (FANTA) indicator guideline and modified for local context[37]. Other parts of the questionnaire were taken from standard DHS questionnaire, and developed by the principal investigators. The questionnaire was administered using local language (Amharic). The content validity of the tool was assessed based on the conceptual framework of the study by relevant professionals, and the reliability of the tool was checked via test-retest method. The tool was pretested on 5% of the total sample outside the study area. During the pre-test, the tool was assessed for its clarity, accuracy of the knowledge measured and comprehensiveness, readability and the optimal time for completing the interview. Modifications were done based on the result. Five data collectors (three clinical mid wives and two laboratory technicians) and one supervisor were recruited. Two days intensive training was given for both data collectors and supervisor by principal investigators prior to the data collection regarding the objective of the study, confidentiality of information, and techniques of interview. To assure quality of the data, the data collection process was followed daily by the supervisor and principal investigators. The dietary diversity (DD) level was assessed using 24-hour recall method. The pregnant women were asked whether they had taken any food from predefined food categories in a day before the survey. Dietary diversity scores were computed based on FAO guidelines[38]. Accordingly, the level of Dietary Diversity Score (DDS) was classified into low (DDS ≤3), medium (DDS of 4 or 5), or high (DDS ≥6).

### Mid upper arm circumference (MUAC) measurement

Mid upper arm circumference was measured halfway between the tip of the shoulder (olecranon process) and the tip of the elbow (acromion process) to the nearest 0.1 cm. The measurement was taken at the mid-point on the relaxed non-dominant hand, without any clothing and with optimal tape tension following the standard instructions and steps[39]. Undernutrition was defined as MUAC less than 22 cm[40].

### Laboratory analysis

#### Blood sample collection and hemoglobin level determination

Venous blood was collected from each pregnant woman, using stainless steel needles and plain tubes (SARSTEDT MonovetteR, Germany). Hemoglobin level was determined using hematological analyzer (Cell Dyn 1800, PD, USA) machine. Anemia was defined as a hemoglobin level of less than 11 g/dl. Anemia was classified into three categories as mild (10–10.9 g/dl), moderate (7–9.9 g/dl) and severe (less than 7 g/dl)[41]. Hemoglobin values were adjusted for altitude according to the formulae recommended by Center for Disease Prevention and Control(CDC)[42].

#### Stool specimen collection and examination

Stool samples were collected from pregnant women using clean, dry and leak-proof cupped plastic container following standard procedures. The stool samples were masked, coded, and processed for parasitological examination. Direct wet-mount and formaldehyde-ether sedimentation method were used for stool examination[43, 44]. The WHO guide for diagnosis of intestinal parasitosis was used as an identification reference[45].

### Data processing and analysis

Data were entered using EPI-INFO version 7 software. Data screening and analysis were carried out using SPSS version 20. Descriptive analysis was done using mean, frequency and percentage. Logistic regression analyses were used in controlling potential confounders. Independent variables significantly associated with the dependent variable in simple regression models were exported to multiple regression models for adjustment. The Odds Ratio (OR) with 95% Confidence Interval (CI) was used to measure the strength of association between anemia and independent variables. The fitness of logistic regression model was assessed using Hosmer-Lemeshow statistic. The collinearity effect was tested using the Variance Inflation Factor (VIF) for all independent variables. The p value of less than 0.05 at 95% CI was considered statistically significant.

### Ethical consideration

Ethical clearance was obtained from ethical review committee of Debre Markos University prior to data collection. Informed written consent was obtained from the study participants before enrolment in the study after the nature of the study was fully explained. Numerical codes instead of names were used in all laboratory forms and questionnaires. Nutrition education was given to all study participants. Pregnant women who were found to have anemia received iron-folate supplementation and counselling according to National treatment guidelines. Pregnant women who were infected with intestinal parasites were given Albendazole tablet.

## Results

### Socio-demographic characteristics of study subjects

All 234 pregnant women, initially planned for the study were volunteered to take part in the study, with a response rate of 100%. The mean age (+/-standard deviation) of the study participants was 26.4 years (+/-4.8 years). The vast majority of the respondents were Amhara in ethnicity (97.9%) and orthodox (92.7%) in religion. Nearly half, (43.2%) of the study participants’ occupations were housewife and one-third, (34.6%) had no formal education (**Table 1**).

**Table 1:** Socio-demographic characteristics of the study participants, Northwest Ethiopia, 2016. (n = 234).

Characteristics		Frequency (n)	Percent (%)
Age(years)	15–24	73	31.2
	25–34	124	53.0
	≥35	37	15.8
Marital status	Married	222	94.9
	Single	12	5.1
Religion	Orthodox	217	92.7
	Muslim	14	6.0
	Protestant	2	0.9
	Catholic	1	0.4
Ethnicity	Amhara	229	97.9
	Oromo	3	1.3
	Tigre	2	0.8
Educational status	No formal education	81	34.6
	Primary education	12	5.2
	High school education	43	18.4
	Certificate and above	98	41.9
Occupation	House wife	101	43.2
	Farmer	17	7.3
	Merchant	29	12.4
	Government employee	73	31.2
	Daily laborer	14	5.9
Family size	≤ 3	149	63.7
	4–6	69	29.5
	>6	16	6.8
Monthly income	Low	46	19.6
	Medium	149	63.7
	High	39	16.7

### Environmental and sanitation factors

**Table 2** summarizes environmental and sanitation characteristics of the study participants. The majority of study participants, 228(97.4%) had toilet facilities. The greater number of study participants, 216(94.7%) use pit latrine. The water source for the majority, 169(72.2%) of study subjects was tap water.

**Table 2:** Environmental and sanitation characteristics of pregnant women attending antenatal care at Debre Markos Referral Hospital, Northwest Ethiopia, 2016.

Characteristics		Frequency (n)	Percent (%)
Source of drinking water	Tab	169	72.2
	Well	10	4.3
	Spring	55	23.5
Possession of toilet facility	Yes	228	97.4
	No	6	2.6
Types of latrine	Pit latrine	216	94.7
	Water flush	7	3.1
	Public	5	2.2

### Reproductive health factors

**Table 3** summarizes reproductive health factors of study participants. More than half, 138 (59%) of the pregnant women were in the third trimester at a time of data collection. The mean gestational age (±SD) of the study participants was 24.2±9.2 weeks. More than half, 137 (58.5%) of the study participants were multi gravida. Among 137 women who gave at least a birth in the previous 5 years of the survey, in 50 (36.5%) of the cases the birth interval was less than the recommended 24 months.

**Table 3:** Reproductive health factors among pregnant women attending antenatal care at Debre Markos Referral Hospital, Northwest Ethiopia, 2016.

Characteristics		Frequency (n)	Percent (%)
Gravida	Primi	97	41.5
	Multi	137	58.5
History of abortion	Yes	13	5.6
	No	221	94.4
History of still birth	Yes	21	9.0
	No	213	91.0
Birth interval(year)	< 1	32	23.4
	1–2	18	13.1
	> 2	87	63.5
Trimester	First	37	15.8
	Second	59	25.2
	Third	138	59
Menstrual character	Regular	192	82.1
	Irregular	42	17.9

### Nutrition related characteristics

The staple diets for the majority, (72.6%) of the study subjects were plant-based foods (made of teff). Daily meal frequency was three times for the majority of the study subjects (62.8%). More than half of study participants, 134 (57.3%) had low dietary diversity score (≤3 food groups). The commonly and frequently consumed food groups were a starchy staple, 100% and legumes, 173(73.9%). Only about one-fifth, 51(21.8%) pregnant women were consumed diet of animal origin in the reference period. More than one-third (35.5%) of the pregnant women were undernourished (MUAC < 22 cm) (**Table 4**).

**Table 4:** Nutrition related Characteristics of pregnant women attending antenatal care at Debre Markos Referral Hospital, Northwest Ethiopia, 2016.

Characteristics		Frequency (n)	Percent (%)
Main staple diet	Teff	170	72.6
	Maize	60	25.6
	Sorghum	2	0.9
	Wheat	2	0.9
Number of meals/day	< 3	17	7.3
	3	147	62.8
	>3	70	29.9
Family food source	Grow their own	72	30.8
	Buy/purchase	161	68.8
	Subsidies/food aid	1	0.4
Nutritional education during pregnancy	Yes	200	85.5
	No	34	14.5
Dietary Diversity	Low	134	57.3
	Medium	53	22.6
	High	47	20.1
Frequency of coffee intake per day	≤ 3 coffee cups (≤ 210ml)	214	91.2
	> 3 coffee cups (>210ml)	20	8.8
MUAC	Undernourished (MUAC < 22 cm)	96	41.0
	Normal(≥22cm)	138	59.0

### Clinical factors

**Fig 1:**
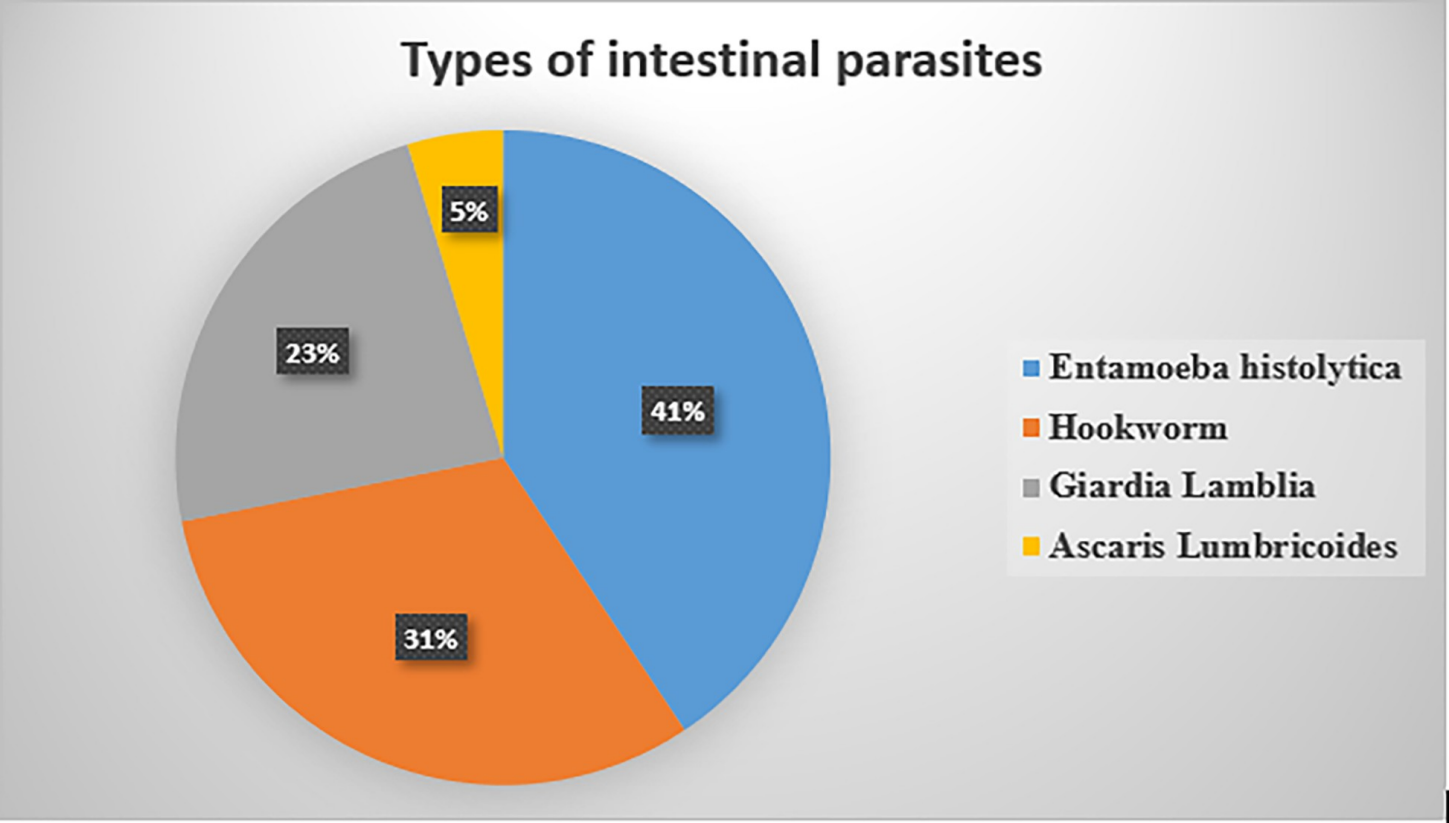
Prevalence and types of intestinal parasites among pregnant women attending antenatal care at Debre Markos Referral Hospital, Northwest Ethiopia, 2016.

More than quarter, 64(27.4%) of pregnant women were infected with one or more intestinal parasites. The most common parasites observed were Entamoeba histolytica 26(40.6%) and Hookworm 20(31.2%) (**Fig 1**). Only 108(46.2%) of pregnant women were taking deworming. Of all respondents, 167 (71.4%) took iron-folate supplement at least once in the preceding four weeks of the survey. However, only 72(43.1%) reported full compliance with the supplement in the reference period. A significant number, 19(8.1%) of the pregnant women were positive for HIV (**Table 5**).

**Table 5:** Clinical factors among pregnant women attending antenatal care at Debre Markos Referral Hospital, Northwest Ethiopia, 2016.

Characteristics		Frequency (n)	Percent (%)
Intestinal Parasite	yes	64	27.4
	No	170	72.6
Deworming	Yes	108	46.2
	No	126	53.8
HIV	Yes	19	8.1
	No	215	91.9
Iron supplement	Yes	167	71.4
	No	67	28.6

### Prevalence of anemia

**Fig 2:**
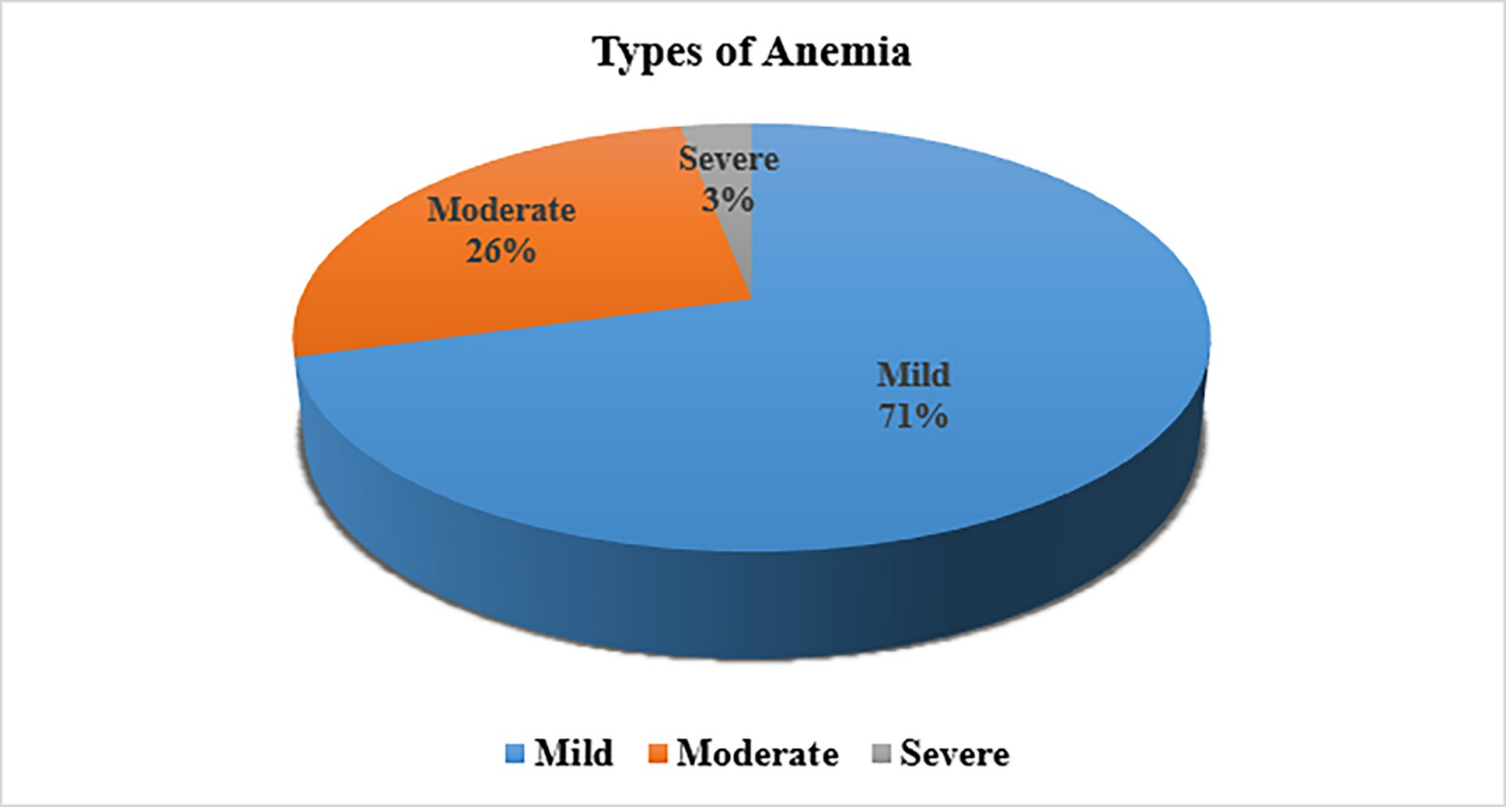
Distribution and severity of anemia among pregnant women attending antenatal care at Debre Markos Referral Hospital, Northwest Ethiopia, 2016.

The study finding showed that the overall prevalence of anemia among pregnant women was 11.5% (95% CI: 8.2%– 14.9%). Among anemic pregnant women, 74.7% were mildly, 26.3% were moderately, and 3% were severely anemic (**Fig 2**). The mean hemoglobin concentration level (±SD) among the study participants was 12.65 (±2.82) g/dl.

### Factors associated with anemia

**Table 6** summarizes factors associated with anemia among pregnant women. A multivariable analysis in a form of logistic regression was employed to identify risk factors of anemia among pregnant women. In the bivariate analysis, anemia was significantly associated with trimester of pregnancy, birth interval, nutrition education during pregnancy, iron supplement, frequency of coffee consumption, MUAC and hookworm infection. The multivariable logistic regression analysis revealed that coffee consumption and hookworm infection were predictors of anemia.

Hookworm infection was found to be significantly associated with anemia after adjustment for potential confounders. Pregnant women who had hookworm infection were two and half times at greater risk of being anemic as compared to pregnant women with no infection [AOR = 2.65; 95% CI (1.48–4.72)]. The study also witnessed significant association between coffee intake and maternal anemia. Coffee intake was associated with lower level of hemoglobin. Compared to pregnant women who consumed ≤ 3 coffee cups per day, the risk of anemia was three times higher among those who consumed > 3 coffee cups per day [AOR = 2.91; 95% CI (1.63–8.78)].

**Table 6:** Factors associated with anemia among pregnant women.

Predictors	Anemia		COR (95%CI)	AOR (95% CI)	P- values
	Yes	No			
**Trimester**					
First	14	23	1		
Second	26	33	0.96(0.33, 2.78)		
Third	76	62	2.06 (0.97, 6.38)		
**Birth interval(year)**					
< 1	18	14	2.16(1.11, 3.38)		
1–2	10	8	0.89(0.29, 2.65)		
>2	51	36	1		
**Nutrition education during pregnancy**					
Yes	109	91	1		
No	21	13	2.63 (0.96, 4.51)		
**Coffee consumption**					
≤ 3 coffee cups (≤ 210ml)	98	116	1		
> 3 coffee cups (>210ml)	13	7	4.03(1.89, 9.95)	2.91(1.63, 8.78)	0.001
**Iron supplement**					
Yes	76	91	1		
No	38	29	1.70 (1.09–3.23)		
**MUAC**					
Normal	80	58	1		
Undernourished	54	42	1.89 (1.16, 3.93)		
**Hookworm infection**					
No	101	113	1	1	
Yes	12	8	3.74 (1.68, 6.83)	2.65 (1.48, 4.72)	0.001

## Discussion

In the current study, 11.5% of pregnant women had anemia based on low hemoglobin levels. According to WHO, anemia is considered of a public health problem if the prevalence of anemia is greater than 5%[4]. Accordingly, with the prevalence of 11.5%, anemia is of public-health concern in the study area. The prevalence determined in this study was considerably lower as compared to other studies conducted in different parts of Ethiopia that were reported 29%[46],31.6%[27], (32.8%)[47], (36.6%)[48], (39.9%)[49], 52%[29] and 56.8%[33]. The prevalence of anemia reported in the current study is also lower than the national prevalence that reported 22%[26]. The current study showed that prevalence of anemia among pregnant women in the country is decreasing as compared to earlier studies. The current study showed that prevalence of anemia among pregnant women in the country is decreasing as compared to earlier studies. This may imply an improvement in maternal nutrition and care during pregnancy. The low prevalence of anemia in the current study may be due to the fact that the interventions employed to address anemia in pregnancy such as; advancements in the quality of antenatal care and every pregnant woman is given iron supplement, deworming, malaria prophylaxis, and mosquito nets. Moreover, traditional variation in feeding habits and cooking a few varieties of food, seasonal difference in data collection, low purchasing power of food, and the role of religious traditions in the Ethiopian diet is very relevant. Further, the study area is one of the districts which has surplus production and a diversified food item produced; as a result, they had a chance to consume of iron rich foods and iron absorption promoters in the diet.

Hookworm infection was significantly associated with an increased risk of anemia in pregnant women. The role of hookworm as risk factor of anemia is consistent with the findings of the past studies[30–32, 34, 46, 48, 50–54]. Hookworm infection may cause anemia by reducing dietary intake, mal-absorption and endogenous nutrient loss. The hookworm also ingests blood by attaching to the mucosa of the upper small intestine, which may cause bleeding within the gastrointestinal tract causing chronic anemia in pregnancy[55–60]. The current study has shown that hookworm contributed to a significant proportion of the anemia in this population, suggesting that all pregnant women should be screened and treated for intestinal parasitic infection during antenatal care visits. Anti-helminthic treatment should be carried out among pregnant women because anti‑helminthic therapy is inexpensive, effective, and safe to administer during pregnancy[61–63].

The study finding indicated a negative association between coffee intake and maternal anemia. Previous study conducted in Costa Rica also supported the finding[64]. Coffee drinking affects iron bioavailability and due to its potency as an inhibitor of absorption is likely to aggravate anemia at times of increased physiological need or when dietary iron intake is precarious[65]. Coffee is known to contain tannin which can potentially interfere with iron absorption[66]. However, empirical evidences are scanty. Further studies with strong study design should be conducted in this direction.

Age, residence, educational status, occupation, family size, monthly income, source of drinking water, latrine availability, gravida, parity, child spacing, trimester of pregnancy, menstrual character, presence of pica, staple diet, frequency of feeding, undernutrition, low dietary diversity, and HIV did not show significant association with anemia. This is may be due to the widespread nature of anemia in the community. A more in-depth understanding of anemia among pregnant women would require further study, using strong study designs with larger sample size. The major limitation of the present study was the cross sectional nature of its design as we can’t establish causal relationships between anemia and the independent variables. Secondly, assessment of dietary intake depends on the 24-hour recall method, which may not accurately reflect their past feeding experience.

## Conclusion

Anemia in pregnancy is of public health concern among pregnant women in the study area. In this study maternal coffee consumption and hookworm infection during pregnancy are key predisposing factors to maternal anemia. Hence, a more comprehensive and community-wide deworming intervention should be performed. All pregnant women coming to antenatal clinics should be screened and treated routinely for intestinal parasitic infection. Anti‑helminthes should be given as prophylaxis to adolescent and young adult women, before their reproductive career. Stool analysis and nutrition intervention should be strongly inculcated into the routine maternity services during antenatal care. We also suggest sustained health education on the dietary intake, access to health care, clean water, hygiene and sanitation for women of reproductive age group particularly, pregnant women. Iron-folate supplementation combined with de-worming will also have affirmative input. The inhibiting effects of coffee on iron absorption can be partially prevail by the concurrent intake of vitamin C rich foods and diet of animal origin. Moreover, pregnant women should limit coffee consumption, and avoid drinking coffee with meals. Causal relationships between maternal coffee consumption during pregnancy and anemia should be further investigated with strong study design.

## Supporting information

S1 Questionnaire (PDF)

## Abbreviations

ANC: Ante Natal Care
CDC: Center of Disease Control
DD: Dietary Diversity
DDS: Dietary Diversity Score
DHS: Demographic and Health Survey
EDHS: Ethiopian Demographic and Health Survey
ETB: Ethiopian Birr
FANTA: Food and Nutrition Technical Assistance
FAO: Food and Agriculture Organization of the United Nations
G/DL: Gram/ Deciliter
Hgb: Hemoglobin
OR: Odds Ratio
SD: Standard Deviation
WHO: World Health Organization
