# Supplementary material for: Anemia and its association with coffee consumption and hookworm infection among pregnant women attending antenatal care at Debre Markos Referral Hospital, Northwest Ethiopia
Source: PLoS One. 2018 Nov 8;13(11):e0206880. doi: 10.1371/journal.pone.0206880 (PMC6224100; doi:10.1371/journal.pone.0206880)
Supplement: S1 Questionnaire — (PDF) [file pone.0206880.s001.pdf]

Annex 3: Consent form English Version

My name is ___________________. I am here to collect information from you about factors associated with Anemia.

I am going to ask you some questions and take blood and stool sample. Your honestly participation in filling questionnaires and giving the required amount of sample will provide us a valid result. Your participation in every aspect of the study is completely voluntary. You may skip any question that you prefer not to answer, but we would appreciate your cooperation. You may also ask me to clarify questions if you don't understand them or can stop the interview at any time. Finally, all the information that you provide for the study is kept completely confidential. Your responses to our questions are identified only by number, never by name. There is no side effects and known health risks related with this research other than a slight discomfort especially during giving blood and it takes only 15 minutes of participation.

Thank you for your participation and listening

Are you willing to participate?

1. Yes 2. No (stop the interview)

Participants Signature __________Date ____/___/____

Data collector Name ___________________Signature __________Date ____/___/____

Supervisor Name _____________________Signature ___________Date____/____/___

Annex 4: Questionnaires

This questionnaire is prepared to collect socio demographic, reproductive and dietary pattern data from pregnant women for the study which aims to assess prevalence and factors associated with anemia among pregnant women in Debre Markos.

Code number: ___________

Chart number: ___________

Part 1: Socio-demographic assessment

S. N	Questions	Response categories	Remarks
101	How old are you?	______ year	
102	What is your religion?	1. Orthodox<br>2. Muslim<br>3. Protestant<br>4. Catholic<br>5. Adventist<br>6. Others specify______	
103	What is your ethnicity	1. Amhara<br>2. Tigire<br>3. Oromo<br>4. Other specify______	
104	What is your current marital status?	1. Single<br>2. Married<br>3. Divorced<br>4. Widowed<br>5. Separated	
105	How many people live in your household?	_______	
106	What is your educational Status?	1. Unable to read and write<br>2. Able to read and write	

		3. Grade 1-4<br>4. Grade 5-8<br>5. Grade 9-10<br>6. Grade 11-12<br>7. Certificate and above	
107	What is your occupation?	1. House wife<br>2. Farmer<br>3. Merchant<br>4. Governmental employee<br>5. Daily labourer<br>6. Other specify_______	
108	What is your monthly income?	__________ ETB	

Part 2: Environmental and Sanitation Factors

S. N	Questions	Response categories	Remarks
201	What is the main source of drinking water to your family?	1. Tap<br>2. Protected wells<br>3. Un protected wells<br>4. Protected Spring<br>5. Un protected Spring<br>4. other specify_______	
202	Do you have latrine?	1. Yes 2. No	

203	If yes to Q 202 what type of latrine facilities do you use in your house?	1. Pit latrine with cover<br>2. Pit latrine without cover<br>4. water flush<br>5. Public<br>6. other specify_______	

Part 3: Clinical Assessment (from study participant and medical chart)

S. N	Questions	Response categories	Remarks
301	Have you ever received iron supplement from a health professional or a health facility?	1. yes<br>2. No	
302	If yes to Q 302 how long did you take iron supplement?	_________	
303	Have you ever had diarrhoea within last two weeks?	1. yes<br>2. No	
304	Have you ever had a blood loss within the past three months?	1. yes<br>2. No	
305	Have you ever had surgical intervention within the past one month?	1. yes<br>2. No	
306	Do you have a history of diabetes mellitus?	1. yes<br>2. No	
307	Do you have a history of cancer?	1. yes<br>2. No	
308	Do you have known chronic organ problem?	1.yes<br>2. No	

309	If yes to Q 411 which organ?	1. Liver<br>2. Kidney<br>3. Heart<br>4. Other specify_____	

Part 4: Reproductive Assessment

S. N	Questions	Response categories	Remarks
401	Do you have live births?	1. Yes 2. No	
402	How many live births do you have ever?	________	
403	Have you ever had still birth?	1. Yes 2. No	
404	If yes to Q 502, how many?	____________	
405	Have you ever had abortion?	1. Yes 2. No	
406	If yes to Q 504, how many?	____________	
407	How long after the first birth did you get the second birth	________	
408	What is your gestational age?	________weeks.	
409	Menstrual character	1.Regular<br>2.Irrigular	

Part 5: Nutrition related factors Assessment

S. N	Questions	Response categories	Remarks
501	What is the main staple diet do you use in your house?	1. Maize<br>2. Teff<br>3. Sorghum<br>3. Millet<br>4. Wheat	

		6. Other (specify)_______	
502	How many times a day do you eat?	________	
503	How does your family meet its food needs?	1. Grow their own<br>2. Buy/purchase<br>3. Subsidies/food aid<br>4. Others: _______________	
504	Have you ever received nutrition education during pregnancy?	1. yes<br>2. No	

Part 6: Behavioral Factors

S. N	Questions	Response categories	Remarks
601	Do you drink coffee?	1.Yes<br>2. No	
602	If yes to Q 801 how many cups do you drink per day?	______cups per day.	
603	Do you drink alcohol or beverages?	1. Yes 2. No	
604	If yes to Q 803 which type of alcohol or beverages?	1. Beer 2. wine<br>3. Tela 4. Teji<br>5. Local areki<br>6.Other (specify)_______	
605	Is there a person who smoke in your house hold or working place?	1.Yes<br>2. No	
606	Do you smoke?	1. Yes 2. No	
607	If yes to Q 807 how many times you smoke per day?	______	

Part 7: Dietary Diversity Assessment

Please describe the foods (meals and snacks) that the respondent ate yesterday during the day and night, whether at home or outside the home.

Breakfast	Snack	Lunch	Snack	Dinner	Snack

When the respondent recall is complete, fill in the food groups based on the information recorded above. For any food groups not mentioned, ask the respondent if a food item from this group was consumed.

S. No	Food group	Examples	Yes=1<br>No=2
701	Starchy staple	corn/maize, rice, wheat, sorghum, teff, millet, bread, enjera, porridge	
702	Dark green leafy vegetables	Spinach, kale, cassava	

703	Vitamin A rich fruits and vegetables	pumpkin, carrots, or sweet potatoes that are orange or dark yellow inside, *red sweet pepper, mango, papaya*	
704	Other fruits and vegetables	Vegetables like tomato, onion, garlic, cabbage, lettuce, and fruits like apple, avocado, banana, lemon, grapes	
705	Organ meat	liver, kidney, heart or other organ meats or blood-based foods	
706	Flesh meat and fish	beef, pork, goat, chicken, or other birds, fish	
707	Eggs	chicken or other bird egg	
708	Legumes/ nuts	beans, peas, lentils, nuts, seeds or foods made from these	
709	Milk and milk products	milk, cheese, yogurt or other milk products	
710	oils and fats	oil, fats or butter added to food or used for cooking	
711	Sweets	sugar, honey, sweetened soda or sweetened juice drinks, sugary foods such as chocolates, candies, cookies and cakes	
712	Spices, condiments, beverages	spices (black pepper, salt), condiments (soy sauce, hot sauce), tea, hard spirits	

Anthropometry

MUAC________________

Laboratory Findings

Intestinal parasite _______________

Hemoglobin ________________

Annex 6. የፈቃደኝነት መጠየቂያ ቅጽ፡ Consent form Amharic version

<u>የጥናቱን አላማ በመረዳት እና በፍቃደኝነት ላይ የተመሰረተ የስምምነት ዉል</u>

ሰላምታ

ስሜ____ይባላል:: እኔ እዚህ እየሰራሁ ያለሁት በሰሜን ምራብ ኢትዮጲያ፣ በደብረማርቆስ ከተማ፤በደብረማርቆስ ሆስፒታል የደም ማነስ ያለባቸውን ነፍሰ-ጡሮች ለማወቅና ሌሎች ተዛማጅ ሁኔታዎችን ለመዳሰስ የተዘጋጀ ጥናት ሲሆን፤ ስለዚህ ይህን ምርምር ለማካሄድ መረጃ በማሳባሰብ ላይ እገኛለሁ::

በመሆኑም የእርስዎ ተሳትፎ ወሳኝ በመሆኑ እርስዎን መሰል ከሆኑ ሰዎች ጋር ተመርጠዋል። ስለዚህ ከእርስዎ ጋር ቃለ መጠየቅ ማድረግ እና የደም ናሙና እፈልጋለሁ።ስለዚህ እርስዎ የሚሰጡን ትክክለኛ መረጃ ለምንፈልገዉ መፍትሄ አየነተኛ ጠቀሜታ አለዉ ብለን ከፍተኛ ግምት ሰጥተነዋል;; ለሚሰጡን መረጃ ስምዎት እና ማንነትዎ አይገለፅም;;

ጥያቄ ካለዎት ይጠይቁኝ____

አሁን በጥናቱ እንዲሳተፉ እጋብዘዎታለሁ:; በአንጻሩ ላለመሳተፉ ከፈለጉ/ ከወሰኑ መብትዎ የተጠበቀ ነዉ::

ለመሳተፍ ፈቃደኛ ነዎት?

አዎ ___________ ቃለ መጠይቅ ይቀጥላል

አይደለም_____________ጥያቄዉን በማቆም አመስግነን እንዲስናበቱ እናደርጋለን::

ቀን_________/________/______________

የተሳታፊው ፊርማ / ዱካ/……………………………………………………………….

የጠያቂዉ ስምና

ፊሪማ____

የተቆጣጣሪው ስምና

ፊርማ____

Annex 7. የአማርኛ መጠይቅ: Amharic version of questionnaire

መጠይቅ:

ይህ መጠይቅ የተዘጋጀዉ በሰሜን ኢትዮጲያ፣ በጎንደር ከተማ፤በጎንደር ዩኒቨርስቲ ሆስፒታል በነፍሰጡር እናቶች ላይ የደም ማነስ መጠንና ተዛማችነት ያላቸውን ምክንያቶችን መረጃ ለመሰብሰብ ነዉ፡፡

መጠይቅ መለያ ቁጥር______________

ካርድ ቁጥር____________________

<u>ክፍል 1፡ አጠቃላይ ማህበራዊና ስነ-ህዝባዊ ሁኔታ</u>

ተ.ቁ	ጥያቄዎች	መልሶች (አማራጮች)	አስተያየት
101	ዕድሜዎት ስንት ነዉ?	________ዓመት	
102	ሃይማኖትዎ ምንድን ነው?	1. ኦርቶዶክስ<br>2. ሙስሊም<br>3. ፕሮቴስታንት<br>4. ካቶልክ<br>5. አድቬንትስት<br>6. ሌላ (ይገለፅ)....................	
103	የየትኛው ብሄረ አባል ነዎት?	1. አማራ<br>2. ትግሬ<br>3. ኦሮሞ<br>4. ሌላ (ይገለፅ).................	
104	የትዳርዎ ሁኔታ ምንድን ነው?	1. ያገባች<br>2. ያላገባች<br>3. የተፋታች<br>4. የሞተባት<br>5. ተለይታ የምትኖር	
105	የቤተሰብዎ ብዛት ምን ያክል ነዉ?	....	

106	የትምህርት ደረጃዎ/ክፍልዎ ስንት ነው?	1. ማንበብ እና መፃፍ የማትችል<br>2. ማንበብ እና መፃፍ የሚትችል<br>3. ከ 1 - 4 ክፍል<br>4. ከ 5 -8 ክፍል<br>5. ከ 9- 10 ክፍል<br>6. ከ 11-12 ክፍል<br>7. ሴርትፍኬትና ከዝያ በላይ	
107	የተሰማሩበት የስራ መስክ/ዋና ስራዎ ምንድን ነው?	1. የቤት እመቤት<br>2. የግብርና ሙያ<br>3. ንግድ<br>4. የመንግስት ተቀጣሪ<br>5. የጉልበት ሰራተኛ<br>6. ሌላ (ይገለፅ) …………………	
108	የቤትሰብዎ የወር ገቢ ምንያክል ነው?	_________ብር	

ክፍል 2፡- የአካባቢ ንጽህና እና ተዛማጅ ሁኔታዎች

201	የመጠጥ ዉሃ በአብዛኛዉ የምታገኙት ከየት ነዉ?	1. የጋራ ቧንቧ/ቦኖ<br>2. የግል ቧንቧ<br>3. የተጠበቀ ጉድጉድ<br>4. ያልተጠበቀ ጉድጉድ<br>5. የተጠበቀ ምንጭ<br>6. ያልተጠበቀ ምንጭ<br>7. ሌላ ካለ ይጠቀስ…………………
202	መፀዳጃ ቤት አለዎት?	1. አወ<br>2. የለም
203	ለ ተ. ቁ 202 መልስዎ አዎ ከሆነ ምን አይነት መፀዳጃ ቤት ነዉ የሚጠቀሙት?	1. ጉድጓድ መጠለያ ያለዉ<br>2. ጉድጓድ መጠለያ የሌለዉ<br>3. በዉሃ የሚሰራ

		4. የህዝብ/የጋራ<br>5. ሌላ ካለ ይጠቀስ...........

ክፍል 3: የበሽታና ተዛማጅ ሁኔታዎች

ተ.ቁ	ጥያቄዎች	መልሶች (አማራጮች)
301	የአይረን ክኒን ወስደዉ ያዉቃሉ?	1. አዎ 2. አላዉቅም
302	ለ ተ. ቁ 402 መልስዎ አዎ ከሆነ ለምን ያህል ጊዜ?	....
303	በዚህ ሁለት ሳምንት ውስጥ አስቀምጦት ያዉቃል?	1. አዎ 2. አያዉቅም
304	በዚህ 3 ወር ዉስጥ የደም መፈሰስ አጋጥመዎታል?	1. አዎ 2. አላጋጠመኝ
305	በዚህ አንድ ወር ዉስጥ ቀዶ ህክምና ተደርጎልዎት ያዉቃል?	1. አዎ 2. አያዉቅም
306	የስኳር በሽታ አለበዎት?	1. አዎ 2. የለብኝም
307	የካንሰር በሽታ አለበዎት?	1. አዎ 2. የለብኝም
308	ለብዙ ጊዜ የቆየ የዉስጥ አካል ክፍል ህመም አለበዎት?	1. አዎ 2. የለብኝም
309	ለ ተ.ቁ 411 መልስዎ አዎ ከሆነ የትኛዉ ነዉ?	1. የጉበት<br>2. የኩላሊት<br>3. የልብ<br>4. ሌላ (ይገለፅ)...................................

ክፍል 4፡ የስነ ተዋልዶ ዳሰሳ

401	ስንት ልጆች አሉኝ?	....
402	ከ28 ሳምንት የእርግዝና ጊዜ በኋላ የፅንስ መቋረጥ (ልጅ ጠፍቶብሽ ያዉቃል)?	1. አዎ<br>2. አያዉቅም
403	ለ ተ.ቁ 502 መልስዎ አዎ ከሆነ ስንት ጊዜ?	....
404	ከ28 ሳምንት የእርግዝና ጊዜ በ ፊት የፅንስ መቋረጥ (ልጅ ጠፍቶብሽ ያዉቃል)?	1. አዎ<br>2. አያዉቅም
405	ለ ተ.ቁ 504 መልስዎ አዎ ከሆነ ስንት ጊዜ?	....
406	በልጆችሽ መካከል ምን ያክል የእድሜ ልዩነት አለ?	_________
407	ከፀነሱ (ነፍሰ ጡር ከሆኑ) ምን ያክል ግዜ ሆነዎት?	_______ሳምንት
408	የወር አበባሽ አመጣጡ እንዴት ነዉ?	1.ግዜዉን ጠብቆ 2. ግዜዉን ሳይጠብቅ

ክፍል 5፡ የስነምግብና ተዛማጅ ዳሰሳ

ተ.ቁ	ጥያቄዎች	መልሶች (አማራጮች)
501	በብዛት በቤት ዉስጥ የሚጠቀሙት የምግብ አይነት ምንድ ነዉ?	1. በቆሎ 2. ጤፍ<br>3. ማሽላ 4. ዳጉሳ<br>5. ስንዴ 6. ሌላ (ይገለፅ)_______
502	በቀን ምን ያህል ጊዜ ይመገባሉ?	_____ጊዜ በቀን
503	የምግብ ፊጆታዎን በአብዛኛዉ የት ነው የሚያገኙት ?	1. እራሳቸው በሚያመረቱት<br>2. ከገቢያ በምገዛት<br>3. ከእረዳታ በሚያገኙት<br>4. ሌላ (ይገለፁ)--------

504	በእርግዝናዎ ጊዜ ስለ አመጋገብዎት ትምህርት (ምክር) አግኝተዉ ያዉቃሉ?	1. አዎ 2. አላዉቅም

ክፍል 6: የባህሪና ተዛማጅ ሁኔታዎች

601	ቡና ይጠጣሉ?	1. አወ 2. አልጠጣም
602	ለተ.ቁ 801 መልስዎ አዎ ከሆነ በቀን ምን ያህል ስኒ ቡና ይጠጣሉ?	_____ የ ቡና ስኒ
603	የአልኮል መጠጦች ይጠጣሉ?	1. አወ 2. አልጠጣም
604	ለተ.ቁ 804 መልስዎ አዎ ከሆነ ምን አይነት የአልኮል መጠጦች ይጠጣሉ?	1. ቢራ 2. ወይን 3. ጠላ 4. ጠጅ 5. የአበሻ አረቂ 6. ሌላ (ይግለፁ)----------
605	በሚሰሩበት ቦታ ወይም ከቤተስብዎት አባላት ውስጥ ሲጋራ የሚያጨስ አለ?	1. አለ 2. የለም
606	ሲጋራ ያጫሳሉ	1. አወ 2. አላጭስም
607	ለ ተ.ቁ 807 መልስዎ አዎ ከሆነ በቀን ሰንት ሲጋራ ያጫሳሉ?	__________

ክፍል 7፡ የምግብ አመጋገብ ልምድ ዳሰሳ

በትላንትናዉ ዕለት በቤት ዉስጥ እና ከቤት ዉጭ የተመገቧቸዉን የምግብ ዐይነቶች እባክዎ በዝርዝር ይንገሩን					
ቁርስ	መክሰስ	ምሳ	መክሰስ	ራት	መክሰስ

መላሾቹ የተመገቡአቸዉን ምግቦች አስታዉሰዉ ከተናገሩ በኋላ በመረጃዉ መሰረት የምግብ አይነቶችን ለይታችሁ አስቀምጡ ።መላሾቹ ተመግበዉ ነገር ግን ያልተናገሩት የምግብ አይነት ካለ ጠይቃችሁ አስቀምጡ ።

S. No	የምግብ አይነት	በአካባቢው የሚገኙ የምግብ ዝርዝር	አወ=1 የለም=2
701	እህል እና የእህል ውጤቶች	በቆሎ፤ ሩዝ፤ ስንዴ፤ ማሽላ፤ ጤፍ፤ ዳጉሳ፤ ፓስታ፤ እንጀራ፤ ቅንጬ፤ ገንፎ	
702	ጥራጥሬ	ባቄላ፤ አተር፤ ሽምብራ፤ ጓያ፤ ኑግ፤ ምስር	
703	አርንጓዴ ቅጠል ያላቸው አትክልቶች	ሰላጣ፤ ካዛቫ፤ ቆስታ	
704	በቫይታሚን የበለፀጉ አትክልቶች እና ፍራፍሬዎች	ዱባ፤ ካሮት፤ቃሪያ፤ ስኳር ድንች፤ ድንች፤ማንጎ፤ ፓፓያ	
705	ሌሎች አትክልቶች	ቲማቲም፤ ቀይ ሽንኩርት፤ነጭ ሽንኩርት፤ የአበሻ ጎምን፤ ጥቅል ጎመን፤ አፕል፤ አቮካዶ፤ ሙዝ፤ ሎሚ፤ የወይን ፍሬ	
706	የአካለት ስጋ	ጉበት፤ ኩላሊት፤ ልብ እና ደም ነክ ምግቦች	
707	ስጋ እና የአሳ ምግቦች	የከብት፤ በግ፤ ፍይል፤ ደሮ፤ ቆቅ፤ ዥግራ፤ አሳ፤ አምባዛ	
708	እንቁላል	የደሮ እንቁላል ጥብስ፤ ቅቅል	
709	ወተት እና የወተት ዉጤቶች	ወተት፤ አይብ፤ እርጎ፤ አጓት፤ ጠለላ	
710	የዘይት እና የቅባት ምግቦች	ዘየት፣ቅቤ የተጨመረባቸው ምግቦች	
711	ጣፋጭ ምግቦች	ስኳር፣ማር፣ጣፋጭ ጭማቂዎች፣ ቸኮሌት፣ከረሜላ፣ኬክ	
712	ቅመማቅመሞች እና መጠጦች	ጨው፣ሻይ፣የለስላሳ መጠጦች	

Anthropometry

MUAC________________

Laboratory Findings

Intestinal parasite _______________

Hemoglobin ________________
